# DNA Methylation Events as Markers for Diagnosis and Management of Acute Myeloid Leukemia and Myelodysplastic Syndrome

**DOI:** 10.1155/2017/5472893

**Published:** 2017-09-06

**Authors:** Geórgia Muccillo Dexheimer, Jayse Alves, Laura Reckziegel, Gabrielle Lazzaretti, Ana Lucia Abujamra

**Affiliations:** ^1^Graduate Program in Biotechnology, UNIVATES, 95914-014 Lajeado, RS, Brazil; ^2^Biological and Health Sciences Center, UNIVATES, 95914-014 Lajeado, RS, Brazil; ^3^Hematology and Hemotherapy Service, Hospital Bruno Born, 95900-010 Lajeado, RS, Brazil

## Abstract

During the onset and progression of hematological malignancies, many changes occur in cellular epigenome, such as hypo- or hypermethylation of CpG islands in promoter regions. DNA methylation is an epigenetic modification that regulates gene expression and is a key event for tumorigenesis. The continuous search for biomarkers that signal early disease, indicate prognosis, and act as therapeutic targets has led to studies investigating the role of DNA in cancer onset and progression. This review focuses on DNA methylation changes as potential biomarkers for diagnosis, prognosis, response to treatment, and early toxicity in acute myeloid leukemia and myelodysplastic syndrome. Here, we report that distinct changes in DNA methylation may alter gene function and drive malignant cellular transformation during several stages of leukemogenesis. Most of these modifications occur at an early stage of disease and may predict myeloid/lymphoid transformation or response to therapy, which justifies its use as a biomarker for disease onset and progression. Methylation patterns, or its dynamic change during treatment, may also be used as markers for patient stratification, disease prognosis, and response to treatment. Further investigations of methylation modifications as therapeutic biomarkers, which may correlate with therapeutic response and/or predict treatment toxicity, are still warranted.

## 1. Introduction

Cancer is generally defined as a group of diseases governed by an accumulation of genetic mutations that are considered to be the major cause of uncontrolled cellular growth [1]. However, epigenetic mechanisms, which alter gene expression without affecting the genetic sequence itself, are also significantly involved in cancer development [2, 3]. Genetic modifications comprise mutations in tumor suppressor genes and oncogenes, both of which skew the balance towards dysregulated cellular proliferation. Epigenetic events are more complex, requiring modifications in chromatin structure or interference with RNA transcripts, and mostly include DNA methylation, histone modifications, nucleosome remodeling, and noncoding RNAs [4]. Thus, during the onset and progression of hematological malignancies, many changes can occur in the cellular epigenome, such as hypomethylation or increases in the methylation of CpG islands in promoter regions of key genes [5].

DNA methylation occurs by the addition of a methyl group (CH_3_) to the 5′-carbon of cytosines that are followed by guanines (CpG sites), resulting in 5-methylcytosine (5-mC). This event is catalyzed by members of the DNMT (DNA methyltransferases) family, mainly DNMT1, DNMT3A, and DNMT3B. DNMT1 is localized in the replication fork during DNA replication, where the new DNA strand is formed. Therefore, this enzyme binds to the daughter strand and methylates it to precisely mimic the original methylation pattern before replication [6, 7]. DNMT3A and DNMT3B present structural and functional similarities. These enzymes are able to introduce methylation into naked DNA, being associated with de novo DNA methylation and, thus, demonstrating an important role in normal development and disease [7, 8]. Methylation of promoter CpG islands usually occurs in or near promoter regions and may disturb the binding of transcription factors. This alone not only contributes to the regulation of gene expression but may also contribute to tumor suppressor gene silencing [9]. Not only that, loss of preserved epigenetic patterns can lead to activation or inhibition of different cellular signaling pathways, which can, invariably, lead to cancer, and it is known that genes that control cell cycle and DNA repair can be mutated or silenced by hypermethylation of their promoter sites [2, 10].

Several studies have already identified mutations in genes that encode crucial epigenetic regulators of gene transcription, such as *IDH1* (isocitrate dehydrogenase 1) and *IDH2* (isocitrate dehydrogenase 2), both of which catalyze the oxidative decarboxylation of isocitrate to 2-oxoglutarate, *TET2* (ten eleven translocation 2) which is an *α*-ketoglutarate-dependent dioxygenase involved in the conversion of 5-methylcytosine (5-mC) to 5-hydroxymethylcytosine (5-hmC) and *DNMT3A*, all of which have been described in hematological malignancies [11–16]. Moreover, DNA methylation is maintained on subsequent cells by DNMT1, responsible for reproducing the parent strand's methylation pattern in the daughter strand [8], and mutations in the DNMT family are frequently described in acute myeloid leukemia (AML) and myelodysplastic syndrome (MDS) [17–19], correlating with poor prognosis [20]. Recently, Spencer and collaborators described that hypomethylation is an initiating event in AML patients with the DNMT3A^R882H^ mutation and DNMT3A-dependent CpG island hypermethylation occurs in consequence of disease progression [21].

The aim of this review is to demonstrate how DNA methylation acts as a potential biomarker for the diagnosis, management, and progression of hematological malignancies, focusing on myelodysplastic syndrome (MDS) and acute myeloid leukemia (AML).

### 1.1. Myelodysplastic Syndrome (MDS) and Acute Myeloid Leukemia (AML)

MDS is a heterogeneous condition of clonal hematopoietic disorders characterized by ineffective erythropoiesis, dysplastic features, chromosomal abnormalities, and increased risk of AML progression. It presents a diverse phenotype, being stratified into low-risk or high-risk disease [22]. Hypomethylating agents (such as 5-azacitidine and 5-aza-2′-deoxycytidine, also known as decitabine), or thalidomide analogues (such as lenalidomide), are already employed for the treatment of MDS. Hematopoietic growth factors, immunosuppressive therapy, and hematopoietic stem cell transplantation are also employed, frequently as second-line therapy [23–25]. The choice of treatment is based on each patient's clinical parameters, such as karyotype, bone marrow blast percentage, and extent of cytopenia, among others [26]. Although bone marrow transplantation is the only choice offering a potentially curative treatment, few patients undergo this procedure because of their advanced age, medical comorbidities, and the limited availability of matching stem cell donors [27]. As supportive therapy, blood and platelet transfusions can be performed, as well as the use of iron chelators and antibiotics [23]. In spite of bone marrow transplantation being the only curative treatment described to date, a randomized phase III trial found that elderly patients with high-risk MDS, complex karyotype, and autosomal monocytes who were treated with decitabine showed higher progression-free survival when compared to patients receiving supportive therapy alone [28].

Decitabine inhibits DNA methylation and, at low doses, may reactivate silenced genes whereas, at high doses, may elicit cytotoxic effects. Studies are conflicting with regard to dose, treatment effectiveness, and patient eligibility, since hypomethylating agents, such as decitabine and 5-azacitidine, are widely used in the clinic even though they yield low complete remission rates, ranging from 15 to 20%. In this context, the treatment eligibility criteria are questioned, as the requirements for patient selection, dose strategy, and treatment duration are not clear, as well as latency and disease response or progression [29]. Dose comparison in decitabine treatments demonstrated more effectiveness at low doses [30]. However, low-dose treatments presented low efficacy and adverse toxic events when compared to treatment with cyclosporine in patients with low- or intermediate-risk MDS [31]. Therefore, a better understanding of response-to-treatment determinants is necessary to improve the therapeutic regimen with hypomethylating agents. In addition to the isolated pathology, other comorbidities should be evaluated in order to recognize which dose each patient should receive [32].

In general, MDS arises from abnormal gene expression, and this expression pattern will define the disease phenotype. Abnormal gene expression stems from different genetic mutations or epigenetic events, which can modify the expression levels of some genes. As an overall rule, these mutations induce cellular growth or inhibit apoptosis and may also block cellular differentiation, resulting in progression to acute leukemia [33]. In fact, the progression of MDS to AML is an example of the multistep theory of carcinogenesis. Kitamura and collaborators presented a new working hypothesis about the molecular bases of hematological malignancies employing the combination of mutations that could influence the phenotype and determine disease. Besides mutations that favor cellular proliferation and that block cellular differentiation, other phenomena in this multistep process were included, such as signal transduction events and epigenetic factors that are associated with dysregulated expression of genes, culminating in cellular immortalization, lack of differentiation, and increased cell survival and growth. Therefore, it is suggested that events that induce cellular immortalization and that favor a less differentiated phenotype are associated with the development of MDS; the addition of events that dysregulate cellular survival and growth provides enough genetic advantages which allows the progression from MDS to AML [34].

AML is a heterogeneous disease, with different molecular signatures, therapeutic responses, and survival rates. It is a result of abnormal blast accumulation in the bone marrow, an event that, eventually, contributes to bone marrow failure. Blasts of the myeloid lineage are also found in peripheral blood at a concentration of approximately 20%. Different DNA methylation signatures have been described as markers for leukemogenesis and prognosis, and these also contribute to the understanding of disease development [35, 36].

Typically, AML treatment is divided into three phases: induction, consolidation, and maintenance. The rationale is to eliminate leukemic cells from the circulation with cytotoxic chemotherapy (induction) and then to eliminate residual leukemic cells from the circulation (consolidation and maintenance) [37]. Anthracyclines and cytarabine arabinoside (AraC) are the main drugs for most of the therapeutic regimens, aiming for complete remission and increased patient survival. Treatments utilizing a combination of these drugs show response rates with complete remission of 70 to 80% for patients under 60 years old [29, 30]. Refractory AML presents a therapeutic challenge, since standard treatment with AraC yields complete response rates of 17 to 20%. One clinical study for refractory AML aimed at achieving better treatment response by combining AraC with lenalidomide but did not present superior results when compared to AraC alone [29]. Moreover, complete remission in AML is generally not sufficient to increase overall survival [31, 32]. This, in part, can be explained by the fact that the presence of mutated genes in AML affects disease progression and prognosis stratifications, making it necessary to understand and validate its effects in order to assist in the clinical management of these patients [35].

Myelosuppression and febrile neutropenia are serious toxic events that arise during treatment and require great attention because of their effect on patient outcome [36, 37]. Although the use of small inhibitory molecules (such as imatinib and dasatinib) and monoclonal antibodies (such as rituximab) allow for longer treatments with lower toxicity rates, studies have already demonstrated that they may lead to serious grade 3 and 4 toxic events [38–40]. Therefore, it is important to establish optimal targets for each disease and to define when and how targeted therapies should be administered in order to establish a better and safer therapeutic regimen [41]. To this effect, determining the methylated genes that are associated with leukemogenesis and disease progression may also be important for selecting new therapeutic targets.

Comorbidities may also influence the therapeutic choices available, to the extent where some cases are considered ineligible for certain therapies because of previous or ongoing toxic events [42]. For patients older than 60 years of age, high-dose chemotherapy is poorly tolerated and treatment is rarely curative. Thus, treatment is directed towards increasing overall survival and quality of life [34]. This poses a challenge, and new approaches are needed in order to improve clinical outcome, contributing not only to better therapeutic responses, overall survival, and disease-free survival but also decreasing toxic events that may be fatal to the patient. Moreover, the development of new therapies demands time and incurs high costs [43]. Therefore, employing a molecular approach may optimize the existing therapeutic regimens, improving response rates, prognosis and, possibly, reducing toxic events.

## 2. Methods

The literature relating DNA methylation and staging/management of MDS and/or AML was reviewed and evaluated, with the goal of verifying which DNA methylation modifications, or changes in gene expression of epigenetic-modulating genes, were most present in disease onset, progression, staging, and toxic events.

The search terms were (biomarker or biomarkers) AND (DNA methylation) AND (acute myeloid leukemia) OR (myelodysplastic syndrome). Eligible literature was identified from PubMed, Science Direct, Web of Science, and Clinical Trial databases, and relevant data were extracted. Unpublished data, comments, letters, and conference proceedings were excluded from this search. A total of 65 articles and clinical trials with methylated genes (or mutations in epigenetic-modulating genes) suggested as marker for diagnosis, management, and prognosis of AML, and/or SMD patients were employed for this review.

## 3. DNA Methylation as an Epigenetic Biomarker

Cancer is characterized by its heterogeneity, given that each patient presents a variable molecular profile, which results in different molecular and physiological characteristics that contribute to development, prognosis, and response to treatment. In this context, the tumor microenvironment plays a fundamental role in which epigenetic components are associated with and contribute to tumorigenesis [44–47]. Epigenetic events, such as DNA methylation, are commonly identified in tumors, and these phenomena may aid in the understanding of the carcinogenic process since it is widely accepted that DNA methylation is related to cancer development and progression [48–51]. Moreover, these changes may be traced back and associated with disease staging and aggressiveness, allowing them to be employed as diagnostic and prognostic biomarkers. For this reason, studies seek to elucidate the interaction between these epigenetic modifications in chromatin remodeling, DNA replication and transcription, and the regulation of genes whose dysregulation is involved in carcinogenesis [52, 53].

Leukemias are a heterogeneous group of malignant neoplasms arising from the myeloid and/or lymphoid lineage, according to the dysplastic cell type, and which affects bone marrow, peripheral blood, and lymphoid tissues [54]. Aberrant epigenetic mutations have been demonstrated in different leukemia subtypes [48, 49, 55], and the number of identified changes is uprising, including genes involved in a plethora of signaling pathways and cellular processes [56, 57]. Association between epigenetic changes, such as DNA methylation, and clinical outcome among leukemia types suggests that these modifications should be explored in order to develop a method that could improve patient stratification [55].

DNA methylation is an extensively studied epigenetic phenomenon, and different gene methylation patterns in tumor cells are used not only as markers for diagnosis but also as therapeutics targets. Different clinical trials have validated the ability of 5-azacytidine, a demethylating agent, in reducing global DNA methylation *in vivo* [58–60]. In this context, inhibitors of DNMT and histone deacetylases (HDAC) demonstrate clinical efficacy in treating hematological malignancies. Fandy and collaborators studied the methylation patterns of *p15^INK4B^* (cyclin-dependent kinase inhibitor 2B), a cell growth regulator; *CDH-1* (cadherin 1), a calcium-dependent cell-cell adhesion molecule; *DAPK-1* (death-associated protein kinase 1), a positive mediator of gamma interferon-induced programmed cell death; and *SOCS-1* (suppressor of cytokine signaling 1), which acts downstream of cytokine receptors participating in the negative feedback of cytokine signaling, in the bone marrow of 30 patients with MDS or AML. After treatment with 5-azacytidine and entinostat, an HDAC inhibitor, reversal of promoter methylation was observed but was not associated with clinical response [58]. In another study, administration of hypomethylating agents, such as decitabine, prior to allogeneic stem cell transplants improved patient outcome, all the while without increasing treatment toxicity in MDS patients [59]. The identification of factors that predict response to therapy could help increase treatment efficacy, while, at the same time, reducing its toxicity. For example, Achille and collaborators investigated global DNA methylation and gene expression of *CDKN2A* (cyclin-dependent kinase inhibitor 2A), *CDKN2B* (cyclin-dependent kinase inhibitor 2B), both regulators of the cell cycle at the G1 checkpoint; *HIC1* (transcriptional repressor 1), a growth regulatory molecule that acts as a tumor repressor; *RARB* (retinoic acid receptor beta), a retinoic acid nuclear receptor which also mediates cellular signalling, growth, and differentiation; *CDH1*; and *APAF1* (apoptotic peptidase activating factor 1), an apoptosis initiator by cleavage of caspase 9, before and during hypomethylating therapy, with the purpose of observing whether early changes could predict clinical response. Although global DNA methylation was not associated with clinical response, decreased *CDKN2A* promoter methylation was observed in patients achieving complete remission, and decreased *CDKN2B*, *RARB*, and *CDH1* promoter methylation was observed in responders [60].

In addition to these applications, DNA methylation can also be used as a biomarker for metastatic tumor screening [61, 62], cancer stage detection [63], malignant progression assessment [64], treatment response [65], and detection of minimal residual disease [66].

The importance of epigenetic modifications can be exemplified by the fact that patients who relapse after first-line therapy, or those stratified as high risk, may present lineage exchange, a phenomenon that occurs when an acute leukemia from the myeloid or lymphoid lineage at diagnosis presents a “switch” to the opposite lineage on relapse [67–70]. This process can be attributed to the original cellular clone, which may present morphological heterogeneity or high plasticity, or to a new leukemic clone. Hypotheses have already been raised in order to explain this event, but its mechanism has not yet been fully elucidated. However, since physiological plasticity is defined as the ability to modify a particular cellular target without altering its genotype, it may be inferred that epigenetic factors participate in mechanisms involved with phenotype regulation mechanisms and with responses to the cellular niche [67–70].

Since DNA methylation can alter gene function and drive malignant cell transformation, and because aberrant methylation modifications usually occur at an early stage of neoplastic development, different DNA methylation patterns may be investigated not only to identify markers for early tumor detection and risk stratification but also to predict treatment response and prognosis [71]. Several studies can be used to illustrate this application: Zhang and colleagues evaluated the clinical relevance of *DLX4* (distal-less homeobox 4) methylation, which plays a role in determining the synthesis of hemoglobin S, in patients diagnosed with MDS. It was found that this gene was significantly hypermethylated in MDS patients when compared to healthy controls. Moreover, patients with hypermethylated *DLX4* had a significantly shorter overall survival compared to patients with hypomethylated *DLX4* [72]. Similarly, *GPX3* (glutathione peroxidase 3) methylation, an enzyme that protects cells from oxidative damage, was identified in the bone marrow of patients diagnosed with MDS and AML, which associated with shorter overall survival compared to patients with unmethylated *GPX3* [73]. Wang and collaborators examined the methylation patterns of Wnt antagonist genes in 144 patients diagnosed with MDS. Survival analysis showed that methylated *sFRP1*, *sFRP4*, and *sFRP5* (secreted frizzled-related protein) were associated with a shorter overall survival. The frizzled-related family has a role in regulating cell growth and differentiation, besides modulating Wnt signaling through direct interaction [74]. In another study, Chaubey and colleagues investigated the effects of the methylation of the supressor of cytokine signalling gene (*SOCS-1*), a negative regulator of the cytokine pathway. A total of 100 patients diagnosed with MDS were evaluated, and methylation was observed in 53% of the cohort. Progression-free survival and median overall survival were shorter in patients in which *SOCS-1* was methylated, in comparison to those with unmethylated *SOCS-1* [75]. Overall, these studies present evidence that the methylation pattern of some genes may influence the course of disease, including with regard to prognosis and survival.

Generally, methylation patterns seem not to be directly related to general clinical data but have demonstrated a direct association to disease classification and stratification. For example, there are reports showing that methylation patterns were not different when compared to gender, age, tumor location, and other clinical parameters, such as white blood cell count [76–78]. Even so, it is important to investigate these methylation patterns across different clinical characteristics in order to observe if there are significant associations or correlations to clinical parameters. Therefore, there is still room to investigate methylation patterns as potential biomarkers for different lineages, as well as for predicting prognosis, response to therapy, and/or toxicity to treatment. Many groups have investigated DNA methylation patterns in these contexts, and their findings are summarized in Table 1.

### 3.1. DNA Methylation as a Biomarker for Diagnosis and Prognosis

Epigenetic modifications, such as DNA methylation, may occur before histopathological changes and, for this reason, may be used as biomarkers for early diagnosis and risk assessment. It is important to note that many types of hematological malignancies are asymptomatic until they reach advanced stages, and, therefore, a thorough characterization of the biomarker is crucial in order for it to be employed for early detection and prediction of tumor progression [114].

Estrogen receptors (ER) regulated by DNA methylation have been reported to play a key role in leukemogenesis. In 40 patients diagnosed with leukemia and evaluated after one year of chemotherapy, it was observed that patients with *ER-α* methylation perceived no symptomatic relief, whereas patients without *ER-α* methylation obtained effective relief with treatment. This data suggest that methylation of *ER-α* could be further investigated as a biomarker for diagnosis and prognosis, since this gene is present in 95% of all evaluated leukemia cases and is related to a lower response to treatments directed towards symptom relief [90].

Methylation of *ID4* (inhibitor of DNA binding 4), a regulator of cell growth, senescence, differentiation, apoptosis, angiogenesis, and neoplastic transformation, was analyzed and suggested as a biomarker for the diagnosis of MDS. Li and collaborators analyzed the methylation status of 100 patients diagnosed with MDS, compared to 31 patients diagnosed with aplastic anemia (AA). *ID4* gene promoter methylation status correlated with clinical parameters in MDS and AA, and bisulfite analysis revealed that gene methylation was higher in patients diagnosed with MDS. Finally, the authors suggest that *ID4* gene promoter methylation could be a causative agent in hematopoietic disorders and, therefore, could be used to distinguish MDS from AA [96]. Similarly, Kang and colleagues investigated *ID4* gene methylation in two patients and in the demethylation-treated MDS cell line (MUTZ1) with bisulfite sequencing PCR. The two MDS patients were treated with decitabine and demonstrated, after treatment, a decrease in methylation. This indicates that this gene may be a biomarker for selection and assessment of effective therapeutic schemes [95].

DNA methylation has also been described as a biomarker for prognosis in hematological malignancies, allowing for a simpler and lower cost analysis than other genetic tests, and also aiding in therapeutic decisions [2, 115–118]. High levels of global DNA methylation are an independent adverse prognostic factor for MDS. Calvo and collaborators, for example, isolated DNA from bone marrow of patients at diagnosis and determined the methylation rate via ELISA. Patients with methylated DNA above 2.73% had a lower overall survival than those with levels below 2.73% and presented a negative trend in terms of leukemia-free survival [119].

Complement C1r (*C1R*) gene methylation, which encodes a protein that is involved in the complement system, has been shown to be a robust, simple, and cost-effective biomarker for prognosis investigation in 194 AML patients. A comparison of *C1R* DNA methylation with healthy donor samples and samples from patients diagnosed with AML showed that patients diagnosed with AML with favorable cytogenetic risk scores had higher methylation in *C1R* and longer overall survival. It was also suggested that DNA methylation of *C1R* might be of independent prognostic relevance; however, further studies must be carried out in order for this to be validated [76].

In another report, Kurtović and collaborators studied samples of newly diagnosed adults with AML, including de novo AML, secondary AML, AML occurring after MDS, and aplastic anemia presenting different cytogenetic patterns. The DNA methylation status of target promoter sequences of *p15* and O-6-methylguanine-DNA methyltransferase (*MGMT*), an enzyme involved in cellular defense against mutagenesis and toxicity from alkylating agents, was analyzed, and 81% of patients presented methylation in at least one of these two genes. It was not possible to prove that *p15* and/or *MGMT* could predict response to therapy and overall survival; however, it was found that AML patients with methylation in both genes or in *p15* alone had a higher frequency of early death and lower frequency of complete remission and presented a trend for shorter overall survival. Moreover, a cluster of abnormalities with adverse prognosis was observed in the group with aberrant methylation of both genes or of *p15* alone [77]. Thus, the methylation pattern of these genes may be used for AML patient stratification. In fact, the *p15* gene was associated with a tumor suppressor role based on its inactivation through hypermethylation of its promoter region in gliomas and leukemias [120]. In addition, this gene often exhibits hypermethylation in its promoter region in adults and children with both myeloid and lymphoid acute leukemia [121, 122].

Also with regard to prognosis, inhibition by methylation of the secreted frizzled-related protein genes *sFRP2* and *sFRP5*, both members of the Wnt pathway, was associated with poor prognosis in normal karyotype AML patients. The Wnt pathway is of great importance, since it plays an important role in the self-renewal of hematopoietic stem cells and in the development of progenitor cells [123]. In another study, Zhou and collaborators investigated the methylation status of the *GPX3* (glutathione peroxidase 3) gene promoter in the bone marrow of 110 MDS patients. Methylation was analyzed by methylation-specific PCR and bisulfite sequencing PCR and was observed in 15% of MDS patients. The methylation rate was higher than those of controls and lower than the methylation rate of AML patients. It was also observed that *GPX3* methylation was associated with older age, higher frequency of *DNMT3A* mutations, and shorter overall survival. The authors conclude that, therefore, *GPX3* methylation in bone marrow could be a marker for adverse prognosis and progression to leukemia in MDS patients [124].

### 3.2. DNA Methylation as a Biomarker for Treatment Response and Toxicity

Both AML and MDS are characterized by an exacerbated proliferation of undifferentiated myeloid cells [29]. Decitabine (5-aza-2′-deoxycytidine) or 5-azacytidine is used to treat these diseases, but there is a chance that more than half of patients will develop resistance to these therapies, leading to worse treatment response [125]. The early identification of whether a patient will respond to treatment is still a major obstacle for achieving clinical success. Evaluations of the clinical course, and subsequent follow-ups, are essential for the safety and efficacy of treatment and for disease remission. Therefore, it is of great importance to identify early markers that may predict which patients will be early responders, late responders, or will not respond at all to treatment.

In a study by Shen and collaborators, it was identified that hypermethylation of *p53*, a vastly studied tumor suppressor gene, and *p73*, which participates in the apoptotic response to DNA damage and, therefore, also acts as a tumor suppressor, correlated strongly with sensitivity to alkylating agents in several cancer cell lines. Six of which were blood- or bone marrow-derived, suggesting that a DNA methylation profile may be useful to identify sensitivity to cancer therapy. However, it should be noted that this study was performed in cultured cell lines and not with patient samples, and, therefore, further studies need to be carried out in order to understand the role of these markers during a patient's clinical course [126].

Another study by Shen and colleagues, with 317 MDS patients, demonstrated that *CDH1*; *CHD13*; *ERα*; *NOR* (oxidored-nitro domain-containing protein isoform 1), a gene that encodes two transcripts and acts as a tumor suppressor; *NPM2* (nucleoplasmin 2), involved in chromatin reprogramming; *OLIG2* (oligodentrocyte lineage transcription factor 2), involved in the chromosomal translocation t(14;21)(q11.2;q22) which is associated with T-cell acute lymphoblastic leukemia; *CDNK2B*; *PGRA* (progesterone receptor A), which functions as transcriptional activator or repressor; and *RIL* (PDZ and LIM domain 4), localized in a region frequently deleted in AML and MDS, were methylated in MDS/AML patients. The methylation pattern before treatment was not associated with clinical response to decitabine. However, methylation reduction after more than four months of treatment correlated with clinical response in 34 patients [127]. In spite of these interesting results, it is important to search for markers that indicate the clinical response before treatment begins or in a shorter time of treatment, in order to aid in choosing the most appropriate therapeutic course for each patient. In a clinical study conducted by Tan and collaborators, it was possible to verify an increase in the acetylation of histones H3 and H4 following treatment with 5-azacitidine combined with panobinostat in AML or MDS patients. The importance of this work stems from the fact that this evaluation was performed utilizing peripheral blood mononuclear cells separated by flow cytometry during the first month of treatment, which is a procedure that could be easily reproduced in other centers [128].

As a matter of fact, genes that have already been related to disease-free survival or disease progression could be reevaluated in peripheral blood in order to corroborate previous findings in bone marrow. For example, the cadherin (CDH) family encodes a calcium-dependent cell-cell adhesion protein, whose loss of function can increase cellular proliferation and invasion, contributing to cancer progression. Other genes, such as *p15^ink4b^* and other tumor suppressor genes, encode cyclin-dependent kinase inhibitors which contribute to cell growth regulation and controls cell cycle progression. Data suggest that methylation of this gene could allow leukemic cells to escape inhibitory signals from the bone marrow. The methylation patterns of these two gene families have already been related to AML progression in MDS patients, and, therefore, could be investigated in peripheral blood as well in order to verify if these results are corroborated [83, 99]. The discovery and validation methylation markers in peripheral blood can be very helpful in investigating response during treatment.

During follow-up, in addition to the therapeutic response, toxic effects are evaluated in order to guarantee the patient's safety. Recent studies have sought to correlate epigenetic regulation of cytokines with tumor development [129, 130]. Moreover, cytokine evaluation was suggested as biomarkers for assessing toxicity during treatment, since they are raised significantly in inflammatory responses. However, they present a short serum half-life and lack toxicity-specific expression [131]. Wang and colleagues assessed serum inflammatory cytokines weekly for 15 weeks in patients with non-small-cell lung cancer during concurrent chemoradiation therapy. An increase in serum IL-6 (interleukin 6), a cytokine that plays a role in inflammation and B-cell maturation, was related to pain, fatigue, disturbed sleep, lack of appetite, and sore throat suggesting a role between proinflammatory cytokine and worsening of symptoms in patients undergoing treatment [132]. With regard to leukemia, Tsapogas and collaborators recently reviewed the role of the cytokine Flt3-ligand (Fms-related tyrosine kinase 3 ligand), which stimulates the proliferation, differentiation, and survival of early hematopoietic cells by activating the FLT3 receptor (Fms-related tyrosine kinase 3), in normal and malignant hematopoiesis [133].

Among the adverse events that may occur during treatment, myelosuppression is the main dose-limiting toxicity and is associated with morbidity and mortality [134, 135]. Febrile neutropenia, or the onset of an infection during neutropenia, represents an emergency and requires administration of broad spectrum antibiotics. These complications may result in reduced dose or interruption of chemotherapy, which compromises patient recovery [101, 136–139]. Moreover, these complications generate high costs, including hospitalization, and may lead to death, demonstrating the importance of its prevention [140, 141].

The understanding of the patient's clinical course, treatment, and risk factors for severe adverse events, such as febrile neutropenia, may allow for preventive actions that reduce the incidence of serious treatment-related complications, all the while reducing the coast of health care [101]. Currently, there are no studies that directly relate and validate changes in epigenetic patterns with the development of toxicity to treatment in hematological malignancies. DNA methylation has already been associated with susceptibility to isoproterenol-induced cardiac pathology in mice. The basal state of the cardiac DNA methylome before and after isoproterenol treatment was compared, and a single-base resolution DNA methylation measurement revealed that treatment decreases global methylation, an event that was associated with heart failure. However, further studies are necessary to investigate this association [142].

Another study with ovarian cancer patients analyzed the methylation in peripheral blood via bisulfite pyrosequencing in different genes during treatment with paclitaxel versus docetaxel. It was observed that higher methylation within the estrogen receptor 1 (*ESR1*) gene was associated with neuropathy on the paclitaxel arm. This was the first cancer study linking DNA methylation in peripheral blood with clinical outcomes, including adverse effects, and suggests that studies evaluating methylation patterns with treatment toxicity in other tumors should also be performed [143]. Another example is the EuroTARGET cohort, a collaborative project that aims to evaluate targeted therapy in renal cell cancer and tumor-related biomarkers for response and toxicity to treatment. Multiplatform “omics,” including the methylome, are being employed to identify biomarkers for toxicity; however, the final data is not yet available [144].

With regard to clinical studies, an ongoing study (NCT02259218; clinicaltrials.gov) aims to identify potential biomarkers that may predict the development of radiation pneumonitis in lung cancer patients and radiation necrosis in brain cancer patients. Metabolic and epigenetic profiles are being studied from blood, urine, and tissue samples in order to find biomarkers that are capable of predicting which patients are more likely to develop adverse effects as a result of radiation treatment [145]. Similar studies should be carried out in order to evaluate biomarkers for toxicity before, during, and after treatment in order to predict early toxic events. It is especially important to investigate these biomarkers in peripheral blood, since samples can be obtained with ease and without need of lengthy preparations for the procedure. This would allow for greater patient safety and drug dose adjustment during treatment, optimizing the therapeutic regimen.

## 4. Conclusion

With regard to MDS and AML, current treatment challenges include choosing the appropriate combination of treatment modalities and chemotherapeutic regimens, since response to therapy is not always achieved. In addition, different adverse effects may occur during treatment because of the toxic effects of most, if not all, chemotherapeutic agents. This seriously delays treatment, affecting the chances of remission, and may directly harm the patient, even leading to death. Moreover, early diagnosis is important in order to increase the potential for a better clinical response during treatment.

Several DNA methylation events affect gene expression and are related to different types of tumors, including hematological malignancies. However, their potential as biomarkers for early diagnosis, stratification, and prediction of treatment response has yet to be more thoroughly evaluated. Studies have demonstrated a significant relationship between DNA methylation patterns and confirmative diagnosis, prognostic potential, and response to treatment. Because changes in DNA methylation are early manifestations and may also act as potential therapeutic targets, the identification of these patterns becomes essential for clinical success. Thus, it is necessary to undertake more studies involving patient samples in order to discover and validate new biomarkers in this field. It is suggested that studies should investigate DNA methylation patterns in peripheral blood samples, in order to optimize not only early diagnosis but also patient management during treatment, allowing for close monitoring of disease progression, adverse events, and response to treatment without the need for bone marrow collection.

## Figures and Tables

**Table 1:** Methylated genes as markers for AML or MDS.

Gene	Disease	Patients (*n*)	Sample type	Associated factors	Ref.
*AWT1*	AML	356	BM/B	Classification of myeloid-derived leukemias. Hypermethylation could monitor the recurrence of disease during remission in patients undergoing allogeneic stem cell transfer.	[79]
*BMI1*	AML/MDS	54	BM/B	DNA methylation was associated with poor prognosis.	[80]
*C1R*	AML	194	B	DNA methylation was associated with the occurrence of specific genomic mutations that are used for risk stratification.	[76]
*CDH*	MDS	60	BM	DNA methylation was associated with poor prognosis and lower complete remission.	[81]
*CDH1*	MDS	317	BM/B	Aberrant DNA methylation predicts overall survival and progression-free survival.	[82]
*CDH1*	MDS	37	BM	Hypermethylation can contribute to the development and poor outcome of disease.	[83]
*CDH13*	MDS	317	BM/B	Aberrant DNA methylation predicts overall survival and progression-free survival.	[82]
*CDKN2B*	MDS	78	BM	DNA methylation was associated with leukemic transformation and disease progression.	[84]
*CDKN2B*	MDS	25	BM	DNA methylation was associated with pathogenesis and prognosis.	[85]
*CEBPA*	AML	181	BM	Methylation was associated with better outcome.	[86]
*CXXC5*	AML	529	BM	Gene was associated with tumor suppressor function in AML and better outcome.	[87]
*DLC-1*	MDS	43	BM/B	DNA methylation was associated with poor prognosis.	[88]
*DLX4*	MDS	103	BM	DNA methylation was associated with poor outcome and shorter overall survival	[72]
*DNMT3A*	LMA	88	B	Methylation was associated with poor prognosis.	[89]
*ERalpha-A*	Leukemia cases with ERalpha-A methylation (95%; 38 of 40)	40	B	Patients with ERalpha-A methylation had no symptomatic relief and patients without this methylation obtained effective relief. ERalpha-A plays a significant role in leukemogenesis.	[90]
*ERalpha-A*	MDS	317	BM/B	Aberrant DNA methylation predicts overall survival and progression-free survival.	[82]
*ERalpha-A*	MDS	37	BM	Hypermethylation can contribute to the development and poor outcome of disease.	[83]
*EVI1*	LMA	476	BM/B	Hipomethylation was associated with poor prognosis.	[91]
*EZH2*	AML/MDS	54	BM/B	DNA methylation was associated with poor prognosis.	[80]
*FHIT*	MDS	—	B	DNA methylation was associated with pathogenesis.	[92]
*GPX3*	MDS	110	BM	DNA methylation was associated with poor prognosis and progression to leukemia in MDS.	[73]
*HIC1*	MDS	37	BM	Hypermethylation can contribute to the development and poor outcome of disease.	[83]
*HIC1*	AML	378	BM/B	Hypermethylation was frequently observed in all types of leukemia and strongly correlated with progression to blast crisis.	[93]
*HOXA5*	AML	378	BM/B	Hypermethylation was frequently observed in all types of leukemia and strongly correlated with progression to blast crisis. Reexpression resulted in the induction of markers of granulocytic differentiation.	[93]
*HRK*	MDS	60	BM	DNA methylation was associated with advanced stage of MDS and progression.	[94]
*ID4*	LMA	212	BM	DNA methylation was associated with shorter overall survival	[73]
*ID4*	MDS	142	BM	DNA methylation was suggested as biomarker for diagnosis.	[95]
*ID4*	MDS	100	BM	DNA methylation was suggested as biomarker for diagnosis.	[96]
*ID4*	AML	14	BM	DNA methylation was suggested as biomarker for minimal residual disease detection.	[66]
*LET-7A-3*	MDS	95	BM	DNA methylation was associated with poor prognosis.	[97]
*MGMT*	AML	21	BM/B	Co-methylation with *p15* gene showed high proportion of leukemic blast cells.	[77]
*MGMT*	AML	30	BM	DNA methylation was suggested as biomarker to predict therapeutic outcome in male AML patients.	[98]
*NOR1*	MDS	317	BM/B	Aberrant DNA methylation predicts overall survival and progression-free survival.	[82]
*NPM2*	MDS	317	BM/B	Aberrant DNA methylation predicts overall survival and progression-free survival.	[82]
*OLIG2*	MDS	317	BM/B	Aberrant DNA methylation predicts overall survival and progression-free survival.	[82]
*p15*	AML	21	BM/B	DNA methylation was associated with higher frequency of early death. Comethylation with *MGMT* gene showed high proportion of leukemic blast cells.	[77]
*p15^INK4b^*	MDS	53	BM	DNA methylation was associated with worse prognosis increasing with disease evolution to AML.	[99]
*p15^INK4b^*	t-MDS; t-AML	81	BM/B	DNA methylation presented a significantly shorter survival and correlated with loss of chromosome arm 7q.	[100]
*p15^INK4b^*	MDS	47	BM	DNA methylation was associated with pediatric disease evolution.	[101]
*p15^INK4b^*	MDS	317	BM/B	Aberrant DNA methylation predicts overall survival and progression-free survival.	[83]
*p15^INK4b^*	MDS	47	BM	DNA methylation was associated with pediatric disease evolution.	[102]
*p21*	MDS	88	BM	DNA methylation could predict clinical outcome.	[103]
*p73*	MDS	88	BM	DNA methylation was associated with poor prognosis in de novo MDS.	[103, 104]
*PcG*	AML	118	BM	DNA methylation was associated with poor prognosis.	[105]
*PGRA*	MDS	317	BM/B	Aberrant DNA methylation predicts overall survival and progression-free survival.	[82]
*PGRB*	MDS	317	BM/B	Aberrant DNA methylation predicts overall survival and progression-free survival.	[82]
*PLA2R1*	MDS	32	B	DNA methylation was associated with disease evolution in MDS and leukemogenesis	[106]
*PLK*	Onco-hematological diseases	ND	BM	Promoter methylation correlates with disease and tumorigenesis in blood neoplasms.	[107]
*PPARD*	AML	344	BM/B	DNA methylation was associated with favorable outcome.	[108]
*PSMD2*	AML	344	BM/B	DNA methylation was associated with favorable outcome.	[108]
*RIL*	MDS	317	BM/B	Aberrant DNA methylation predicts overall survival and progression-free survival.	[82]
*RING1*	AML/MDS	54	BM/B	DNA methylation was associated with poor prognosis.	[80]
*sFRP1*	MDS	144	BM	DNA methylation was associated with worse overall survival and poor prognosis	[74]
*sFRP2*	AML	72	BM/B	DNA methylation was associated with increased risk of relapse and risk of death, predicting adverse clinical outcome in patients with normal karyotypes.	[109]
*sFRP2*	MDS	144	BM	DNA methylation was associated with worse overall survival and poor prognosis	[74]
*sFRP5*	AML	72	BM/B	DNA methylation was associated with increased risk of relapse and risk of death, predicting adverse clinical outcome in patients with normal karyotypes.	[109]
*sFRP5*	MDS	144	BM	DNA methylation was associated with worse overall survival and poor prognosis	[74]
*SOCS-1*	MDS	100	B	DNA methylation was associated with disease progression and poor survival	[75]
*SOX17*	MDS	164	BM	DNA methylation was associated with poor prognosis.	[110]
*TERTpro/Ex1*	AML	43	BM	Hypermethylation was associated with inferior patient survival.	[111]
*TERTpro/Ex1*	AML/MDS	33	BM/B	DNA methylation was associated with poor prognosis and inferior patient survival.	[111]
*VTRNA1–3*	MDS	140	BM	DNA methylation was associated with poor outcome.	[112]
*XPNPEP*	AML	344	BM/B	DNA methylation was associated with unfavorable outcome.	[108]
*ZO-1*	MDS	ND	BM	DNA methylation was associated with disease progression.	[113]

AML: acute myeloid leukemia; B: peripheral blood; BM: bone marrow; MDS: myelodysplastic syndrome; ND: not declared; t-AML: therapy-related acute myeloid leukemia; t-MDS: therapy-related myelodysplastic syndrome.
